# Exploring the Binding Mechanism of NRG1–ERBB3 Complex and Discovery of Potent Natural Products to Reduce Diabetes-Assisted Breast Cancer Progression

**DOI:** 10.1007/s12539-023-00566-y

**Published:** 2023-06-30

**Authors:** Sulaiman K. Marafie, Eman Alshawaf, Mohamed Abu-Farha, Thangavel Alphonse Thanaraj, Dong-Qing Wei, Fahd Al-Mulla, Abbas Khan, Jehad Abubaker, Anwar Mohammad

**Affiliations:** 1 Department of Biochemistry and Molecular Biology, Dasman Diabetes Institute, Kuwait City, Kuwait; 2 Special Service Facilities, Dasman Diabetes Institute, Kuwait City, Kuwait; 3 Department of Genetics and Bioinformatics, Dasman Diabetes Institute, Kuwait City, Kuwait; 4 Department of Bioinformatics and Biological Statistics, School of Life Sciences and Biotechnology, Shanghai Jiao Tong University, Shanghai, 200240 People’s Republic of China

**Keywords:** NRG1, ERBB3, Breast cancer, Diabetes mellitus, Molecular dynamic simulations, MM/GBSA

## Abstract

Diabetes mellitus significantly contributes to breast cancer progression, where hyperglycemia upregulates specific genes, leading to more aggressive tumor growth. In patients with BC that develop diabetes, neuregulin 1 (*NRG1*) and epidermal growth factor receptor 3 (*ERBB3*) overexpression exacerbate tumor growth and progression. Since the interaction between NRG1 and ERBB3 is critical for tumor growth, understanding the molecular mechanisms underlying NRG1–ERBB3 complex formation is essential for elucidating diabetes-assisted breast cancer progression. However, the key residues forming the NRG1–ERBB3 complex remain unknown. Here, we substituted specific residues in NRG1 with alanine and studied its interactions with ERBB3 using computational structural biology tools. We further screened the South African natural compounds database to target the complex’s interface residues to discover potential inhibitors. The conformational stability and dynamic features of NRG1–WT, –H2A, –L3A, and –K35A complexed with ERBB3 were subjected to 400 ns molecular dynamics simulations. The free binding energies of all NRG1–ERBB3 complexes were calculated using the molecular mechanics-generalized Born surface area (MM/GBSA). The H2 and L3 alanine substitutions caused a loss of interaction with ERBB3 residue D73, weakening the interaction with ERBB3. Screening 1300 natural compounds identified four (SANC00643, SANC00824, SANC00975, and SANC00335) with the best potential to inhibit ERRB3-NRG1 coupling. The binding free energies for each complex were − 48.55 kcal/mol for SANC00643, − 47.68 kcal/mol for SANC00824, − 46.04 kcal/mol for SANC00975, and − 45.29 kcal/mol for SANC00335, showing their overall stronger binding with ERBB3 than NRG1 and their potential to act as ERBB3-NRG1 complex inhibitors. In conclusion, this complex may represent a residue-specific drug target to inhibit BC progression.

**Graphical abstract:**

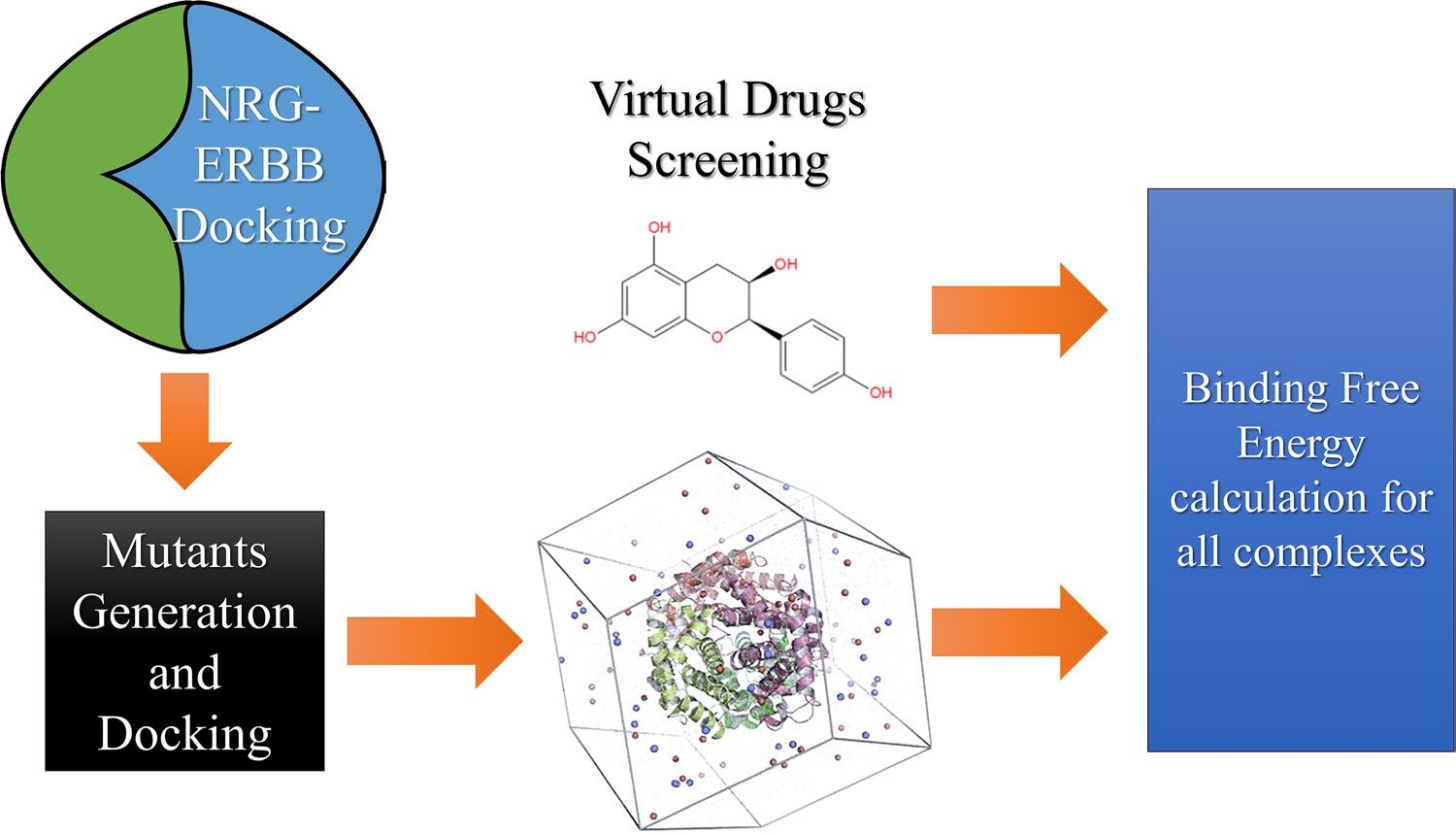

**Supplementary Information:**

The online version contains supplementary material available at 10.1007/s12539-023-00566-y.

## Introduction

Numerous factors contribute to cancer progression, including diabetes mellitus (DM) [1–3]. Hyperglycemia results in a more aggressive tumor growth behavior caused by the upregulation of specific genes involved in signaling pathways that target tumor progression. Studies have shown that the neuregulin 1 (NRG1) and human epidermal growth factor (EGF) receptor (HER) signaling pathway promotes carcinogenesis and the pathogenesis of multiple human cancers, including breast cancer (BC) [4–7]. Consequently, patients with DM and BC showed *NRG1* overexpression that potentiates ERBB3 signaling through an autocrine pathway [6, 8].

Neuregulins (NRGs) are signaling proteins expressed in the nervous system, heart, and breast as ligands for the erb-b2 receptor tyrosine kinase (ERBB) family (ERBB1–4): EGF receptor (EGFR/ERBB1), ERBB2/HER2, ERBB3/HER3, and ERBB4/HER4 [9]. The ERBB family comprises an extracellular N-terminal ligand-binding domain connected to an enzymatically active cytoplasmic domain. ERBB3 is a 185 kDa protein comprising an extracellular domain (residues 20–630), a transmembrane domain (residues 642–666), a juxtamembrane domain (residues 667–709), a tyrosine kinase domain (residues 709–965) with an activation loop (residues 830–890), and a C-terminal tail (residues 990–1342) [10]. NRG1 interacts with ERBBs to activate downstream signaling pathways such as phosphoinositide 3-kinase (PI3K), nuclear factor kappa B (NF-κB), and extracellular signal-regulated kinase (ERK) [11, 12]. The NRG1–ERBB3 signaling pathway has a well-documented role in carcinogenesis and the pathogenesis of multiple human cancers, including BC [4, 6, 7, 12]. Park et al. performed comparative genome-wide mapping of hyperglycemia-specific open chromatin regions, identifying *NRG1* as a critical tumor cell growth regulator in BC. *NRG1*’s importance was supported by its upregulated mRNA expression (20-fold) in tumors of patients with DM [8]. Elevated *NRG1* levels co-existed with *ERBB3* overexpression, contributing to BC progression.

NRGs are encoded by four genes (NRG1-4) and have mitogenic and pro-apoptotic characteristics, giving them dual oncogenic and tumor suppressor functions [13, 14]. *NRG1* expression results in six proteins and 31 splice variants with various biological processes and properties, with all isoforms forming an extracellular EGF-like domain [13, 15]. NRG1’s structure [16, 17] comprises a three-stranded β-sheet at the N-terminus and a two-stranded β-sheet at the C-terminus. Previous reports showed the importance of the NRG1-N-terminal domain’s receptor specificity by substituting residues 1–5, resulting in a bifunctional molecule capable of binding EGF and ERBB2, ERBB3, and ERBB4 [18]. To identify the key residues crucial for NRG1-N-terminal interaction, Jones et al. performed alanine scanning of the entire N-terminal region and measured its interaction with ERBB3 and ERBB4. Notably, alanine substitutions to residues His2, Leu3, and Lys35 significantly affected ERBB3 binding. Interestingly, the His2 and Leu3 to alanine substitutions reduced the binding affinity to ERBB3 but not ERBB4 [19].

Initial studies did not identify specific residues playing a crucial role in the NRG1–ERBB3 binding receptor interface [19], further exacerbating BC progression in patients with DM [4]. However, alanine scanning only identified the key NRG1 residues interacting with ERRB3. Therefore, understanding the key residues forming the NRG1–ERBB3 complex would be critical in developing selective receptor antagonists to block NRG1–ERBB3 interactions and prevent BC tumor progression. Computational tools to decipher the interaction mechanism are indispensable and have been used by various studies [20–22]. Therefore, using computational structural biology tools, this study elucidated the structural impact of substituting ERBB3-interacting NRG1 residues with alanine at the atomic level. The structural stability and conformational dynamic features of NRG1-wildtype (WT), –H2A, –L3A, and –K35A complexed with ERBB3 were tested by running 400 ns molecular dynamic (MD) simulations for each complex. In addition, to extract the free binding energies of each NRG1–ERBB3 complex, we used the molecular mechanics-generalized Born surface area (MM/GBSA). The H2 and L3 alanine substitutions caused a loss of interaction with ERBB3 at residue D73, weakening its interaction with ERBB3. Therefore, this may represent a residue-specific drug target for inhibiting BC progression.

## Materials and Methods

### Structure Retrieval, Modeling, and Preparation

The 3D structural coordinates of ERBB3 and NRG1 were retrieved from https://www.rcsb.org/ Protein Data Bank (PDB) using accession IDs 4LEO and 1HAE, respectively [23]. The structures were subjected to topological defects and missing residues. Chimera’s embedded Modeller program was used to model the missing residues [24, 25]. The model determines the 3D structure of a query protein by satisfying spatial restraints. The loops are usually defined using the de novo approach, while multiple other parameters, such as multiple sequence alignments, are used to refine the structure prediction. Then, the structures were minimized using Chimera’s conjugated gradient and steepest descent algorithms [24, 25]. The finally relaxed and minimized structures were then processed for further analysis.

### Macromolecular Docking and Interface Analysis

The restrained docking of ERBB3 with NRG1–WT, His2Ala, Leu3Ala, and Lys35Ala structures was performed using the HADDOCK server (REF). HADDOCK uses a docking process that encodes information from known or projected protein interfaces in ambiguous interaction restraints (AIRs). In addition, HADDOCK from experimental data by cryo-electromagnetic maps and nuclear magnetic resonance (NMR) residual dipolar couplings and pseudo-contact shifts unambiguous distance restraints are defined. The protonation state was set to default, which was left as “autohis = true.” The *Z*-positioning restraints were set to default as experimental restraint. The surface contacts restraint was set as “surfrest = true,” while the dihedral angle restraints were set to default [26]. The ERBB3-NRG1 complexes with the lowest *Z* score and the most significant structure size were selected for MD simulations analysis. The best docking complexes were then subjected to in silico alanine mutagenesis using the mCSM-PPI2 web server [27], where the residues may help recognize and process ERBB3-NRG1 signaling. The impact of each residue’s substitution was defined and subjected to all-atoms MD simulation.

### NRG1–ERBB3 Complex Dynamics

The structural-dynamic features of the WT complex and three substitutions (His2Ala, Leu3Ala, and Lys35Ala) were each explored through a 400-ns all-atoms MD simulation. We used the AMBER20 simulation package with the FF19SB model to perform simulations [28, 29]. Each system was solvated in an optimal point-charge solvation box and followed the neutralization by adding counter ions. The system was minimized using 6000 and 3000 steps of the steepest descent and conjugate gradient algorithms. System heating at 300K and equilibration were performed. Finally, each complex’s production runs lasted 400 ns. Simulation trajectories were generated and analyzed using AMBER’s CPPTRAJ and PTRAJ modules [30].

### Binding-Free Energy Estimation

Total binding free energy estimation of NRG1–WT, –H2A, –L3A, and –K35A in complex with ERBB3 were computed using the MMGBSA.py script [31]. The MM/GBSA method was used, and various energy terms were determined, such as electrostatic energy, van der Waals energy (vdW), and polar and non-polar solvation energies. Various studies have used this method to understand binding energy [32–34]. Each complex’s net binding free energy was obtained with the equation:

$$=\boldsymbol{ }{\Delta G}_{\mathrm{complex binding energy}}-\left[ {\Delta G}_{\mathrm{receptor binding energy}}+ {\Delta G}_{\mathrm{ligand binding energy}}\right].$$

Each of the above net binding energy components can be split as follows:

$$G= {G}_{\mathrm{bonded}}+ {G}_{\mathrm{vdW}}+{G}_{\mathrm{polar solvation energy}}+{G}_{n\mathrm{on}-\mathrm{polar solvation energy}.}$$

The entropic computation was not performed because it is computationally costly and highly operation prone to significant errors [35].

### Molecular Screening of Natural Compounds Against the Interface Residues

To identify potential inhibitors targeting ERBB3’s interface residues to abrogate the binding with NRG1, virtual drugs screening of the South African Natural Compounds Database (SANCDB) was screened. ERBB3’s interacting residues required for interactions with NRG1 were used to generate a grid with an XYZ size of 0.75, − 1.59, and − 5.09. Furthermore, Autodock vina was used to screen the whole database [36, 37]. A python script rearranged the compounds based on their docking scores. The best four compounds were subjected to free energy calculation using the fastDRH online web tool [38].

## Results and Discussion

### NRG1–ERBB3 Structure

**Fig. 1:**
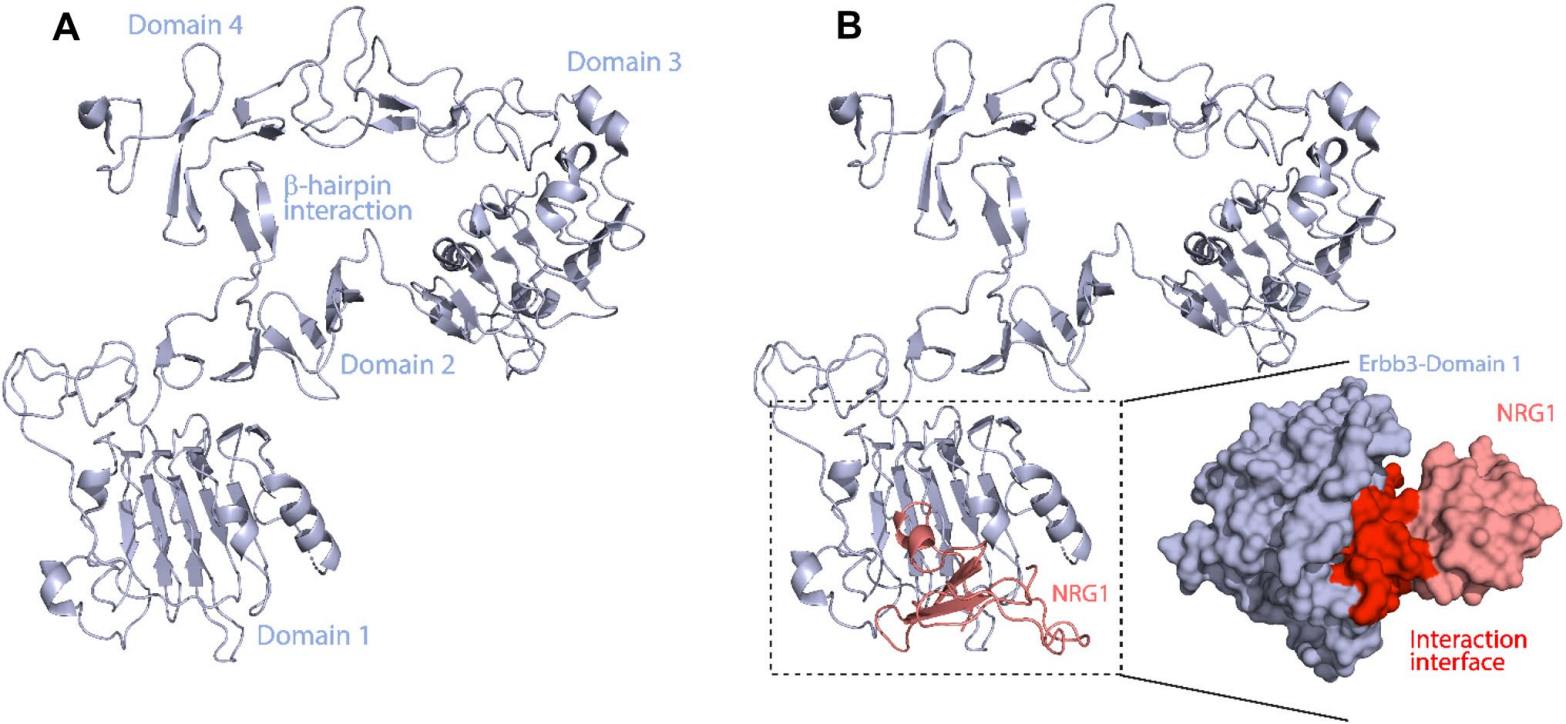
**A** Structure of ERBB3’s extracellular domain (PDB ID: 4LEO; light blue). **B** ERBB3’s extracellular domain interaction with NRG1 (PDB ID: 1HAE; salmon). The binding interface between NRG1 and ERBB3 in the space-fill 3D structure

**Fig. 2:**
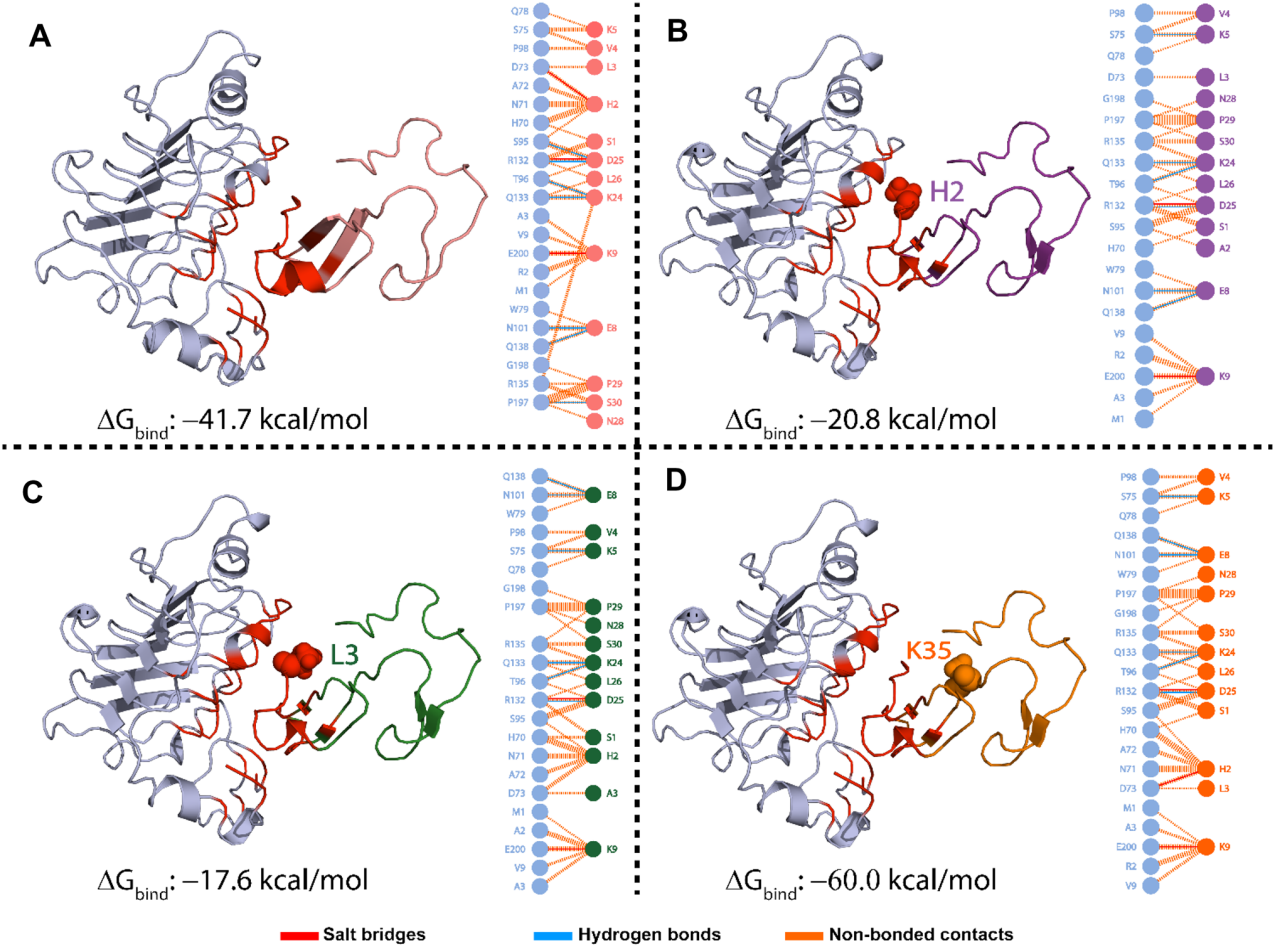
Docking representation of the NRG1 complex with ERBB3 and a 2D schematic diagram of the bonding interactions between key residues in the NRG1–ERBB3 interface: **A** NRG1–WT, **B** NRG1–H2A, **C** NRG-L2A, and **D** NRG1–K35A. The balls represent the mutated residues in the NRG1 structure and define the mutation site

The ERBB3 extracellular domain comprises four subdomains (I–IV), with domains I (ligand-binding domain) and III being leucine-rich β-helical folds in the structure (Fig. 1A). In contrast, domains II (C1) and IV (C2) are cysteine-rich and involved in ligand-induced receptor dimerization with ERBB2 [12] (Fig. 1A). NRG1’s N-terminal domain interacts with ERBB3 extracellular domain I, initiating the ERBB3 signaling pathway (Fig. 1B). Therefore, this study used the X-ray crystal structure of ERBB3’s extracellular domain (PDB ID: 4LEO) [12] for the interaction analysis with NRG1’s NMR solution structure (PDB ID: 1HAE) (Fig. 1B) [17]. Domain 1 of the ERBB3 extracellular domain (residues 1–200) was extracted from the 4LEO file, and the missing residues were modeled using Chimera’s embedded Modeller program [24]. The modeled domain I structure was validated against the X-ray crystal structure using PyMOL with a root-mean-square deviation (RMSD) of 0.973 Å. Furthermore, the NRG1–WT, –H2A, –L3A, and –K35A structures were docked to domain 1 on the HADDOCK server to perform 400 ns MD simulations. In addition, the interaction interface residues between NRG1 and domain 1 are depicted using a script InterfaceResidues.py and PDBsum protein-protein analysis using the ligplot algorithm [39] (Fig. 2).

### NRG1–ERBB3 Interactions

Initial studies showed that substituting specific NRG1 residues influenced its interaction with the ERBB3 extracellular domain. In particular, residues H2, L3, and K35 showed the greatest impacts on the NRG1–ERBB3 interaction, affecting the ERBB3 signaling pathway. Therefore, the intermolecular interactions of ERBB3’s extracellular domain I with NRG1–H2A, –L3A, and –K35 were compared with NRG1–WT using the HADDOCK protein–protein docking server. The NRG1–WT interaction with ERBB3’s extracellular domain I formed seven hydrogen bonds (H-bonds) and three salt bridges (Fig. 2A). NRG1 residue H2 formed a salt bridge with ERBB3 residue D73 and hydrophobic interactions with residues S95, H70, N71, and A72. In comparison, L3 formed a hydrophobic interaction with residue D73 since leucine is a hydrophobic amino acid and mainly forms hydrophobic interactions through its side chain [40].

NRG1–H2A’s interaction with ERBB3 resulted in six H-bonds and two salt bridges. The H2A substitution resulted in the loss of the salt bridge with ERBB3 residue D73 (Fig. 2B). Furthermore, it resulted in the loss of four hydrophobic bonds in the binding interface with ERBB3, which may be essential for the NRG1–ERBB3 interaction and activity. The NRG1–L3A–ERBB3 complex had six H-bonds and two salt bridges (Fig. 2C). Consequently, the L3A substitution caused the loss of the hydrophobic interaction with ERBB3 residue D73, which also lost a salt bridge with the H2A substitution. Therefore, the L3A substitution caused a more dynamic NRG1 structure that affected the interaction of both H2 and L3 with ERBB3. These results have shown the importance of ERBB3 residue D73 in its interaction interface with NRG1. The NRG1–K35A substitution resulted in six H-bonds and three salt bridges with ERBB3 (Fig. 2D). While K35 is not part of the NRG1–ERBB3 interaction interface, its substitution may affect NRG1’s intermolecular interactions. Such intermolecular changes can result in a structural shift in NRG1, influencing its binding to ERBB3.

### MD Simulations of NRG1–ERBB3 Complexes

**Fig. 3:**
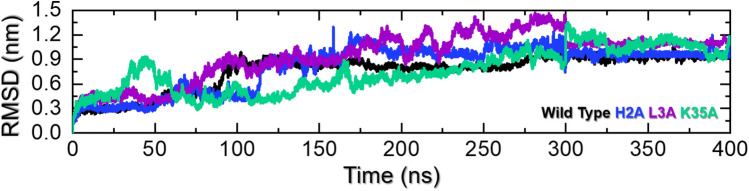
RMSDs for ERBB3 complexed with NRG1–WT (black), NRG1–H2A (blue), NRG1–L3A (purple), and NRG1–K35A (green). The *x*-axis shows simulation time in ns, and the *y*-axis depicts the RMSD in nm

We estimated the structural and conformational changes of NRG1–WT, –H2A, –L3A, and –K35A complexes with ERBB3 using a 400-ns simulation performed with the AMBER 20 package. The RMSD trajectories of alpha-carbon (Cα)-atoms demonstrate the dynamic stability and convergence of the NRG1–ERBB3 complexes (Fig. 3). The calculated root-mean-square fluctuations (RMSF) of the Cα-atoms showed each complex’s residual flexibility (Fig. 5). Furthermore, the stability and compactness of NRG1–ERBB3 complexes were measured by radius of gyration (Rg), where a stable Rg value indicates the protein’s correct folding (Fig. 6). The impact of each substitution in the NRG1 interface was assessed using total binding free energy (Δ*G*) based on the MM/GBSA method (Fig. 2 and Table 1).

**Table 1:** The binding free energy variables (kcal/mol) from MM/GBSA of NRG1–WT, –H2A, –L3A, and –K35A complexed with ERBB3

Complex	MM/GBSA	0–50 ns	51–100 ns	101–150 ns	151–200 ns	201–300 ns	301–350 ns	351–400 ns	Average
WT	vdW	− 72.01	− 44.34	− 52.68	− 64.26	− 59.34	− 56.11	− 55.98	− **57.82**
	Electrostatic	− 248.90	− 146.4	− 320.82	− 314.16	− 327.26	− 321.47	− 318.69	− **285.39**
	GB	296.73	182.84	332.93	336.84	334.59	342.25	336.77	**308.99**
	SA	− 9.92	− 5.57	− 8.43	− 9.42	− 9.11	− 9.20	− 9.14	− **8.68**
	Δ*G*	** − 34.11**	− **13.47**	− **49.01**	− **50.99**	− **61.12**	− **44.53**	− **47.04**	− **42.90**
His2Ala	vdW	− 76.77	− 37.17	− 1.62	− 32.32	− 43.16	− 34.47	− 39.19	− 37.81
	Electrostatic	− 258.16	− 273.38	− 99.66	− 133.27	− 183.86	− 185.65	− 175.9	− 187.13
	GB	309.09	306.03	99.41	155.77	193.45	184.77	175.53	203.44
	SA	− 10.03	− 5.28	− 0.74	− 4.35	− 7.99	− 6.98	− 7.53	− 6.13
	Δ*G*	** − 35.88**	− **9.80**	− **2.61**	− **14.18**	− **41.56**	− **42.33**	− **47.09**	− **27.64**
Leu3Ala	vdW	− 34.97	− 36.80	− 37.47	− 35.93	− 39.22	− 38.32	− 36.66	− **37.05**
	Electrostatic	− 173.54	− 242.63	− 207.28	− 132.02	− 154.66	− 149.72	− 157.33	− **173.88**
	GB	209.62	267.23	237.00	157.66	160.45	161.55	160.49	**193.43**
	SA	− 4.23	− 5.11	− 4.96	− 4.75	− 6.47	− 6.39	− 6.11	− **5.43**
	Δ*G*	** − 3.12**	** − 17.31**	− **12.72**	− **15.04**	− **39.90**	− **32.88**	− **39.61**	− **22.94**
Lys35Ala	vdW	− 83.96	− 67.82	− 100.33	− 67.49	− 70.90	− 68.51	− 65.23	− **74.89**
	Electrostatic	− 231.90	− 203.55	− 465.60	− 391.40	− 250.58	− 238.51	− 240.74	− **288.90**
	GB	276.94	243.81	483.32	402.86	281.98	270.47	268.19	**318.22**
	SA	− 12.59	− 9.18	− 14.63	− 9.96	− 8.99	− 7.79	− 8.36	− **10.21**
	Δ*G*	** − 51.51**	** − 36.74**	− **97.24**	− **65.99**	− **48.49**	− **44.34**	− **46.14**	− **55.78**

Bold shows the total binding free energy

#### Root Mean Square Deviations of NRG1–ERBB3 complexes

The RMSD of the NRG1–WT complex with ERBB3 (Fig. 3) showed a deviation from 0 to 0.3 nm in the initial ten ns. The RMSD remained stable at 3 nm from 10 to 50 ns before converging to 5 nm from 50 to 90 ns. Furthermore, the NRG1–WT-ERBB3 complex showed an atomic fluctuation from 90 to 110 ns, increasing the RMSD from 0.5 to 0.8 nm. Subsequently, the NRG1–WT-ERBB3 complex presented an RMSD between 0.8 and 0.9 nm from 110 to 300 ns, indicating a stable system. During the final 100 ns (301–400 ns), the structure showed a stable uniform RMSD, indicating that the complex had already reached stability. NRG1–WT-ERBB3 complex stability further substantiates the enhanced ERBB3 pathway activity upon NRG1 interaction in patients with DM and BC [4].

**Fig. 4:**
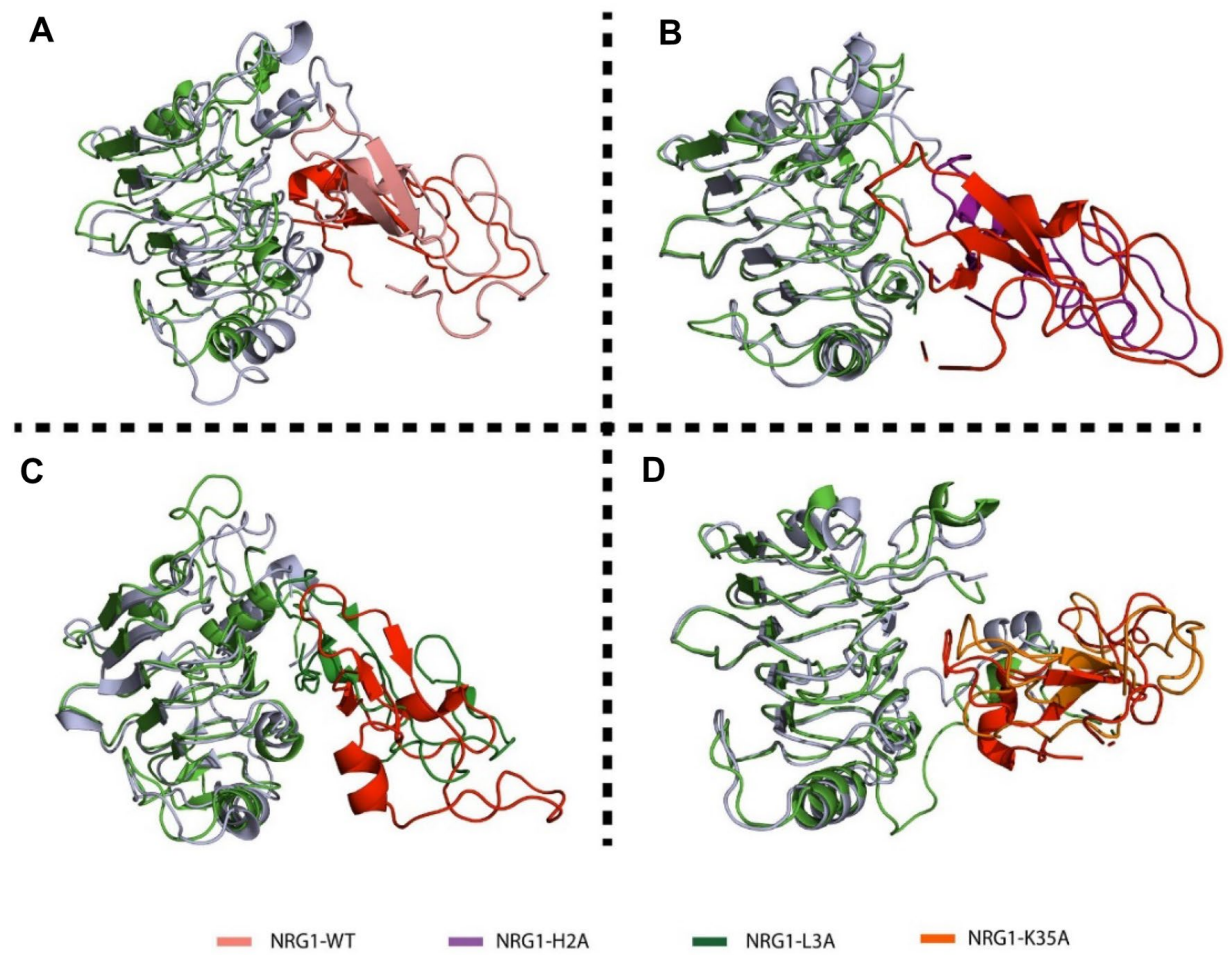
Structural alignment of ERBB3 complexed with **A** NRG1–WT, **B** NRG1–H2A, **C** NRG1–L3A, and **D** NRG1–K35A at the beginning (0 ns) and end (300 ns) of the MD simulations

In contrast, the NRG1–H2A and –L3A interactions with ERBB3 indicated an unstable system (Fig. 4B, C). The NRG1–H2A complex with ERBB3 (Fig. 3) showed an increase in RMSD to 0.3 nm in the initial five ns, where the system stabilized for 50 ns. Subsequently, the NRG1–H2A-ERBB3 complex converged to 0.7 nm from 50 to 75 ns, after which the RMSD decreased to 0.44 nm at 110 ns. From 110 ns, the NRG1–H2A-ERBB3 showed a highly dynamic and unstable system, with RMSDs increasing to 1.0 nm and fluctuating to 1.2 nm until the end of the 300 ns simulation. After 300 ns, the system RMSD abruptly decreased and showed a flattened uniform RMSD, indicating stability. The system converged with the WT system after 300 ns, showing that both had attained a similar dynamic configuration (Fig. 3). Similarly, the NRG1–L3A complex with ERBB3 showed high dynamic fluctuations causing high structural perturbation, with unstable RMSDs from 0 to 100 ns. Subsequently, the NRG1–L3A–ERBB3 complex converged, and RMSDs increased from 2 to 14 nm during the 400 ns simulation.

The instability observed in NRG1–H2A and –L3A complexes with ERBB3 could reflect the loss of the interaction with ERBB3 residue D73. The loss of the salt bridge between H2 and D73 can influence the interacting stability between NRG1 and ERBB3, resulting in a less stable system. In addition, histidines play a vital role in protein stability [41, 42], and this substitution may have influenced NRG1’s internal structural dynamics, affecting its interaction with ERBB3. The instability is shown in the NRG1–L3A-ERBB3 complex results from the loss of the hydrophobic interaction between L3 and ERBB3 residue D73. NRG1–H2A and –L3A substitutions affected the interaction with ERBB3 residue D73, potentially affecting ERBB3’s downstream activity. Like the H2A complex, the system RMSD stabilized after 300 ns and converged with the WT system. These findings are consistent with experimental results that showed NRG1 H2 and L3 substitution affected ERBB3 function [19].

NRG1–WT–ERBB3 complex formation triggers a downstream pathway activating PI3K/protein kinase B (AKT) signaling mechanisms, inducing drug resistance in cancer [43]. The AKT signaling pathway regulates several cell cycle modulators, such as cyclin-dependent kinase inhibitor 1B (CDKN1B/p27^Kip1^), that induce cell cycle progression and modulate several apoptotic pathways [44, 45]. AKT signaling pathways stimulate mammalian target of rapamycin (mTOR) activity that activates ribosomal protein S6 kinase B2 (RPS6KB2/p70S6K) and eukaryotic translation initiation factor 4E-binding protein 1 (EIF4EBP1/4E-BP1) to regulate G1-S phase cell cycle transition [46]. Therefore, the weaker interactions between NRG1 and ERBB3 after substituting NRG1 residues H2 and L3 identify them as potential drug targets to disrupt NRG1–ERBB3 interactions and interrupt AKT signaling by inhibiting its phosphorylation, leading to growth arrest and apoptosis [47].

Compared to the NRG1–H2A and –L3A complexes with ERBB3, the NRG1–K35A–ERBB3 complex showed a very stable system (Fig. 3). It converged for the first 45 ns, reaching an RMSD of 1.0 nm. Subsequently, the system stabilized after 60 ns, with the average RMSD remaining at 6 nm for the remainder of the 300 ns simulation, with a slight convergence at 120 ns. The NRG1–K35A substitution resulted in a stable complex with ERBB3, increasing downstream ERBB3 activity. However, K35A substitution results contrast with Jones et al. [19], who observed a decrease in ERBB3 interaction and subsequent activity with NRG1–K35A. The increased RMSD after 300 ns may be due to removing the positively charged lysine residue, potentially resulting in charge-charge repulsions with other positively charged residues in the moiety [48, 49].

We demonstrate the effects caused by these mutations on the NRG1–ERBB3 complex by comparing their structural deviations from native structures after 300-ns simulations (Fig. 4). The NRG1–WT structure deviated, and a secondary structure transition was observed. In addition, the NRG1 structure moved further towards the binding interface, while the ERRB3 structure achieved more structure packing, causing structural deviations. Despite the structural rearrangement observed between ERBB3 and NRG1–H2A in the complex, a gap appeared between the two interacting proteins (Fig. 4). Furthermore, similar observations were made with the NRG1–L3A–RBB3 complex despite the loop’s movements, indicating that NRG1’s structure had moved away from the ERBB3 interface (Fig. 4). The NRG1–K35A interaction with ERBB3 showed a conserved structural arrangement and minor NRG1 deviation towards the interface’s downward side. Therefore, the H2A and L3A substitutions destabilize the binding by destabilizing NRG1 and pushing it away from the ERBB3 interface.

#### RMSFs of NRG1–ERBB3 Complexes

**Fig. 5:**
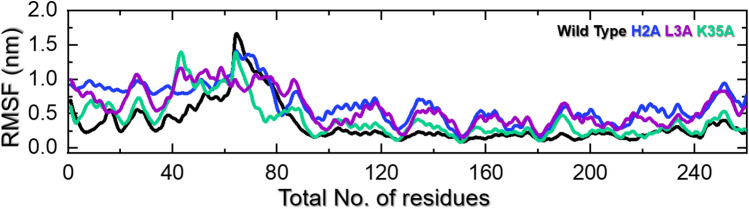
RMSFs for ERBB3 complexed with NRG1–WT (black), NRG1–H2A (blue), NRG1–L3A (purple), and NRG1–K35A (green). The *x*-axis shows the residue number, and the *y*-axis depicts the RMSF in nm

The RMSF of the Cα-atoms from the 400 ns simulations for NRG1–WT, –H2A, –L3A, and –K35A complexed with ERBB3 are shown in Fig. 5. The NRG1–WT, –H2A, –L3A, and –K35A complexes with ERBB showed similar high residual fluctuations, averaging 0.4–0.8 nm for the first 1–80 amino acids. However, the NRG1–H2A–ERBB3 and NRG1–L3A–ERBB3 complexes showed higher fluctuations between residues 100 and 300 than the NRG1–WT–ERBB3 and NRG1–K35A–ERBB3 complexes. As a result of their substitutions, the NRG1–H2A and –L3A structures lost the interaction with residue ERBB3–D73, causing conformational changes that resulted in a weaker interaction with ERBB3 and higher structural fluctuation. H2 displacement resulted in a salt bridge with D73, which can provide favorable free energy binding in protein-protein interactions. When unfulfilled, the isolated charge may not form a salt bridge, substantially destabilizing the binding of two interacting proteins [50]. The NRG1–WT–ERBB3 complex showed higher residue flexibility for the region between residues 61 and 80. However, removing the charged residue (K35) resulted in a more stable NRG1 complex with ERBB3 (Fig. 5). Removing charged residues has been shown to increase protein stability. Studies on the ubiquitin protein have shown that removing lysine residues increased protein stability by 6.8 kJ/mol [48]. Interestingly, the results with NRG1–WT and –K35A were consistent. While their behavior also aligns with the docking scores, their free energy calculations show a distinct pattern due to the K35A mutation, though it is far from the binding interface.

#### Rg of the NRG1–ERBB3 Complexes

**Fig. 6:**
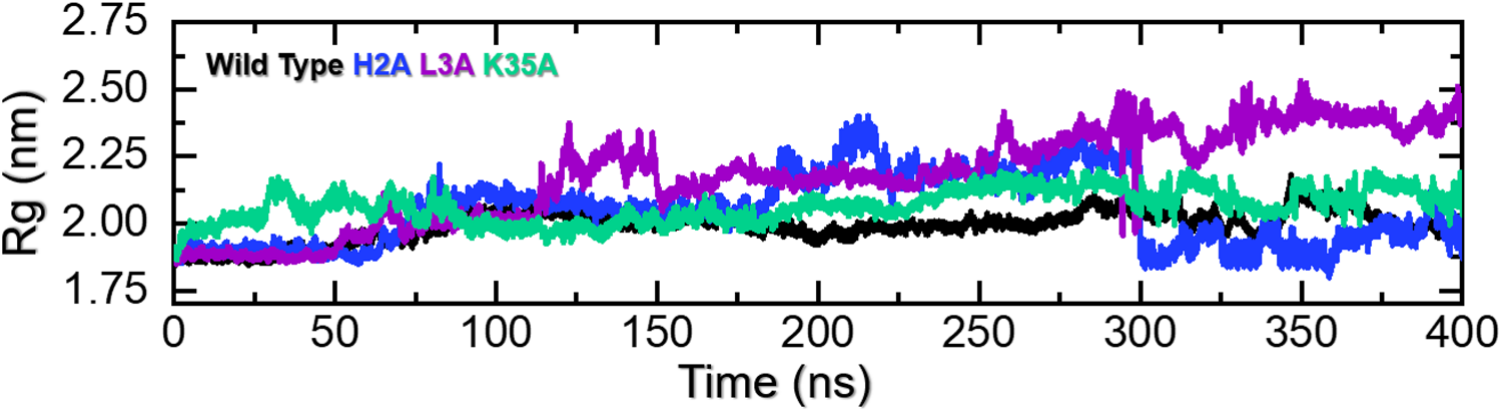
Rgs for ERBB3 complexed with NRG1–WT (black), NRG1–H2A (blue), NRG1–L3A (purple), and NRG1–K35A (green). The *x*-axis shows time in ns, and the y-axis shows Rg in nm

Each complex’s structural compactness was evaluated using Rg as a function of time (Fig. 6). The NRG1–WT interaction with ERBB3 remained more compact with a mean Rg of 1.92 nm, while the NRG1–H2A and -L2A interactions with ERBB3 (Fig. 6) showed greater Rg fluctuations. The NRG1–H2A-ERBB3 complex’s compactness diminished after 100 ns, fluctuating between an RMSD of 2.1–2.4 nm from 120 to 300 ns. The NRG1–L2A–ERBB3 complex was compact, with an average Rg of 1.9 nm for the initial 60 ns. Then, the complex started to lose compactness, fluctuating between 2.2 and 2.5 nm from 175 ns until the end of the simulation. The NRG1–K35A–ERBB3 complex’s structure initially remained less compact until 80 ns, after which it gained compactness with an average Rg of ~ 2.0 nm for the remainder of the 300 ns simulation. After 300 ns, a similar pattern of compactness was observed for all complexes, with RMSDs indicating stability.

Therefore, the NRG1–WT–ERBB3 complex’s compactness, as indicated by the Rg results, corroborates its stability indicated by RMSDs and RMSFs. In comparison, NRG1–H2A and –L3A complexes with ERBB3 lost compactness, resulting from the loss of essential bonds with ERBB3. The increased protein size is due to the loop movement of ERBB3, resulting in the loss of some contact due to an increase in the distance of key residues. However, the NRG1–K35A–ERBB3 complex remained more compact, consistent with the RMSD and RMSF data, indicating that removing the charged lysine residue results in a more compact and stable structure.

#### Binding Energies of NRG1 to ERBB3

MD simulation analyses confirmed that the NRG1 amino acid substitutions caused structural remodeling and affected ERBB3 binding. To further confirm their influence, ΔG was calculated for NRG1–WT, –H2A, –L3A, and –K35A complexed with ERBB3 using the MM/GBSA method in different time intervals [51] (Table 1). NRG1–WT showed an average Δ*G* of − 42.90 kcal/mol with ERBB3, a tighter interaction than NRG1–H2A (Δ*G* = − 27.64 kcal/mol) and NRG1–L3A (*ΔG* = − 22.94 kcal/mol) with ERBB3. The loss of the essential salt bridge between NRG1–H2 and ERBB3–D73 would be significant for the NRG1–ERBB3 complex. The NRG1–H2A-ERBB3 complex initially (0–50 ns) had a tighter Δ*G* (− 35.88 kcal/mol) than the NRG1–WT–ERBB3 complex (− 34.11 kcal/mol). However, as the system converged, the Δ*G* for the NRG1–H2A–ERBB3 reduced to − 15.62 kcal/mol. Furthermore, the NRG1–H2–ERBB3 complex showed weaker electrostatic energies (− 187.13 kcal/mol) than the NRG1–WT–ERBB3 complex (− 285.39 kcal/mol), further indicating that losing the electrostatic interaction reduced protein-protein binding energies.

Similarly, the NRG1–L3A formed an unstable complex with ERBB3, showing a Δ*G* of − 3.12 kcal/mol from 0 to 50 ns. Then, the complex fluctuated with a final Δ*G* of − 12.04 kcal/mol. The loss of the hydrophobic interaction with D73 caused a weaker Δ*G* between ERBB3 and NRG1–L3A than NRG1–WT. The loss of hydrophobic interactions affected the ordering of the molecules involved in the protein-protein interactions, decreasing binding energy [50]. Furthermore, some studies indicated that a loss of hydrophobicity results in weaker surface area interaction (SA) [52]. The average SA was weaker for the NRG1–L3A–ERBB3 complex (− 5.43 kcal/mol) than the NRG1–WT-ERBB3 complex (− 8.68 kcal/mol). An average Δ*G* for the NRG1–L3A–ERBB3 complex after 400 ns was − 22.94 kcal/mol. The tighter overall binding of NRG1–WT to ERBB3 might indicate that elevated NRG1 concentrations exacerbate BC conditions by forming a tight complex. The weaker interactions between ERBB3 and NRG1–H2A and NRG1–L3A reduced ERBB3 pathway activity. Hydrophobic interactions, H-bonds, and salt bridges all play significant roles in the binding energies of protein-protein interactions. An H-bond or salt bridge induces a more favorable binding energy between two interacting proteins. Therefore, a missing H-bond or salt bridge affects protein-protein interaction stability, resulting in weak binding energy [50]. Importantly, these results suggest that these two residues could act as therapeutic hotspots for discovering novel drugs for treating DM-facilitated BC progression.

The NRG1–K35A substitution showed an average **Δ**G of − 60.0 kcal/mol with ERBB3, tighter than the NRG1–WT interaction with ERBB3 (− 41.74 kcal/mol) [53]. The positively charged K35 residue is near another positively charged residue (Q37; 11.0 nm), and its removal may have resulted in the removal of charge-charge repulsion, stabilizing the NRG1 structure. Charge repulsion is a long-range effect, defined as 1/*r* where *r* demonstrates the inter-atomic distance between two charged groups [49, 54]. Consequently, side chains with similar charges in the same region can have a weak charge-charge repulsion effect on one other. The NRG1–K35A–ERBB3 complex’s stability is further supported by its stronger vdW (− 74.89 kcal/mol), electrostatic (− 288.90 kcal/mol), generalized Born (GB; 318.22 kcal/mol), and SA (− 10.21 kcal/mol) energies than the NRG1–WT–ERBB3 complex (Table 1). The average **Δ**G for the NRG1–K35A–ERBB3 complex after 400 ns was − 55.78 kcal/mol. The K35A substitution resulted in a tight and stable NRG1–ERBB3 complex, contrasting with the findings of Jones et al., who found that it significantly reduced ERBB3 binding [19]. Consequently, NRG1–K35A’s tighter binding to ERBB3 might promote tumor progression, contributing to a more aggressive BC.

We further subjected the NRG-1WT structure to druggable site identification based on these findings. We found that the interface residues act as the drug-binding site and could be targeted using novel drugs. The identified putative binding site is shown in Supplementary Figure S1. However, further analysis and studies are required to corroborate the NRG1–K35A substitution’s effect. Each complex attained stability during the last 100 ns, displaying minimum fluctuation in the binding energy parameters, indicating reliable results.

## Targeting the Interface Site with Natural Products

**Fig. 7:**
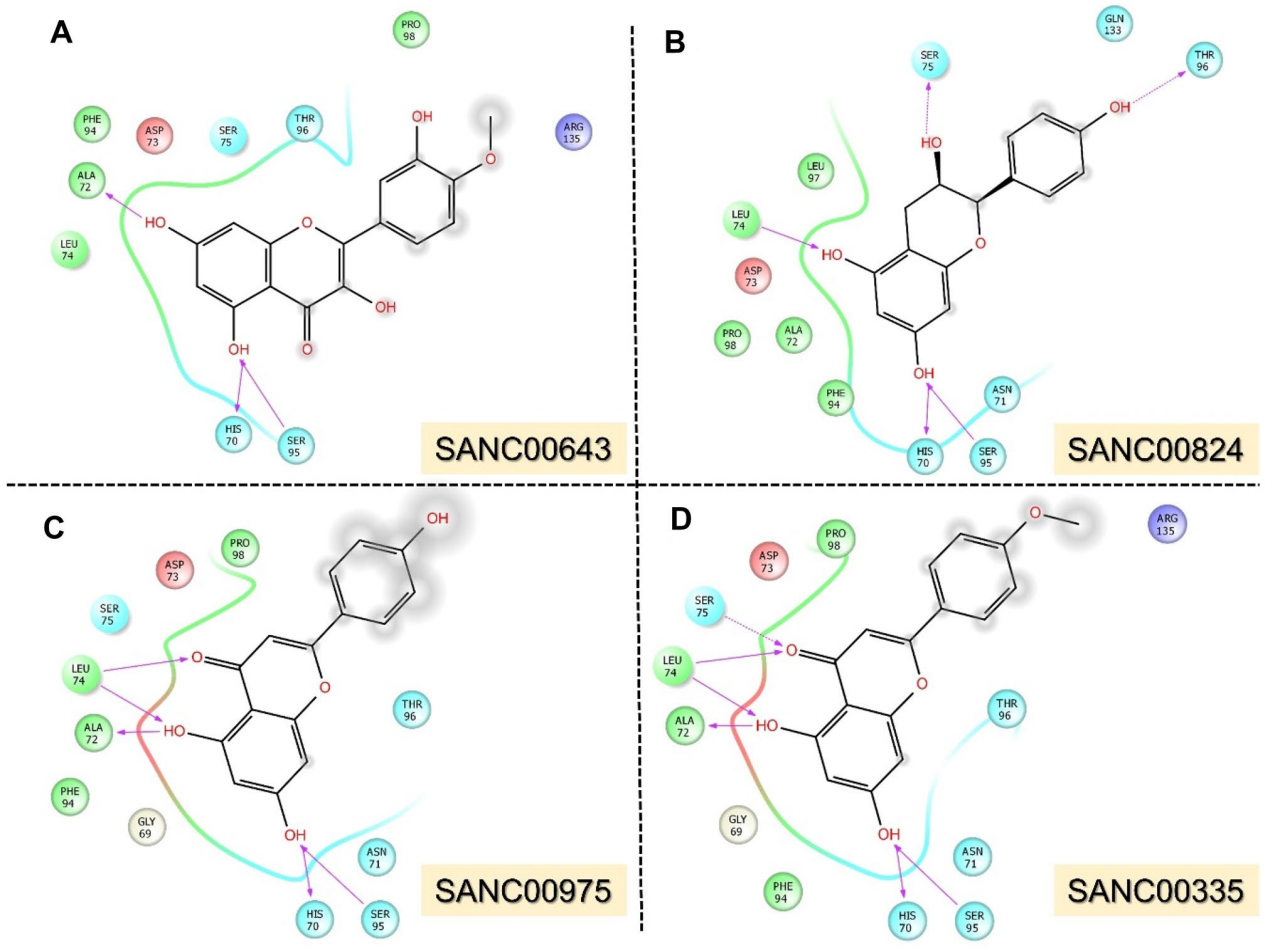
Interaction mode of the best SANCDB hits. Binding modes are shown for **A** SANC00643, **B** SANC00824, **C** SANC00975, and **D** SANC00335

Screening of 1300 natural compounds identified four (SANC00643, SANC00824, SANC00975, and SANC00335) that could potentially inhibit ERRB3-NRG1 binding. SANC00643, a flavonoid known as Afzelechin, had the most significant docking score of − 8.42 kcal/mol, establishing hydrogen bonds with His70, Ala72, and Ser95. Its interaction pattern is shown in Fig. 7A. SANC00824, known as Apigenin, is shown in Fig. 7B and had a docking score of − 8.40 kcal/mol, establishing interactions with His70, Leu74, Ser75, Ser95, and Thr96. SANC00975, known as Buddleoflavonol, had a docking score of − 8.31 kcal/mol, establishing interactions with His70, Ala72, Leu74, and Ser95. Its binding mode is shown in Fig. 7C. Finally, SANC00335, known as Tamarixetin, established hydrogen bonds with His70, Ala72, Leu74, Ser75, and Ser95 and had a docking score of − 8.16 kcal/mol. The ΔG was − 48.55 kcal/mol for SANC00643, − 47.68 kcal/mol for SANC00824, − 46.04 kcal/mol for SANC00975, and − 45.29 kcal/mol for SANC00335. These findings show the stronger overall binding of these compounds with ERBB3 than NRG1, highlighting their potential to act as ERBB3-NRG1 complex inhibitors. These compounds further need experimental validation to confirm the inhibitory potential of these molecules that could aid the treatment of diabetes assisted breast cancer. The database IDs, 2D structures, docking scores, and Δ*G*s for these compounds are provided in Table 2. Schrodinger Maestro’s free academic version was used for 2D interaction visualization only.

**Table 2:** The database IDs, 2D structures, docking scores, and Δ*G*s for the four compounds

Database ID	2D structure	Docking score	Δ*G*
SANC00643		− 8.42	− 48.55
SANC00824		− 8.40	− 47.68
SANC00975		− 8.31	− 46.04
SANC00335		− 8.16	− 45.29

## Conclusions

The NRG1–ERBB3 interaction plays a vital role in BC progression, where the overexpression of both proteins in patients with DM further exacerbates tumor development. Therefore, the NRG1–ERBB3 interaction interface is an interesting target for anti-tumor drug development to block tumor progression. Here, we studied the impact of alanine substitutions on NRG1’s interaction with ERBB3. We used biomolecular docking and MD simulation approaches to compare the binding affinities of NRG1–WT, –H2A, –L3A, and –K35A with ERBB3. The MD simulation and MM/GBSA results support the importance of H2 and L3 residues in NRG1–ERBB3 interactions, with the NRG1–H2A and NRG1–L3A substitutions significantly decreasing their binding energy. The loss of vital intermolecular interactions with residue ERBB3–D73 weakened the NRG1–ERBB3 interaction. Furthermore, the screening of 1300 natural compounds identified four (SANC00643, SANC00824, SANC00975, and SANC00335) as potential ERRB3-NRG1 binding inhibitors. Therefore, our findings highlight NRG1 H2 and L3 residue’s interaction with ERBB3–D73 and their potential importance in developing novel NRG1–ERBB3 inhibitors for treating and inhibiting tumor progression in DMs-facilitated BC.

## Supplementary Information

Below is the link to the electronic supplementary material.

Supplementary file1 (DOCX 622 KB)

## Data Availability

Data will be made available on reasonable request.
