# Supplementary material for: Exploring the Binding Mechanism of NRG1–ERBB3 Complex and Discovery of Potent Natural Products to Reduce Diabetes-Assisted Breast Cancer Progression
Source: Interdiscip Sci. 2023 Jun 30;15(3):452–64. doi: 10.1007/s12539-023-00566-y (PMC10374477; doi:10.1007/s12539-023-00566-y)
Supplement: Supplementary file 1 — Supplementary file1 (DOCX 622 KB) [file 12539_2023_566_MOESM1_ESM.docx]

Structural Analysis to Understand the Molecular Mechanisms of the NRG1-ERBB3 Complex and its Role in Diabetes-Assisted Breast Cancer Progression

Supplementary Information


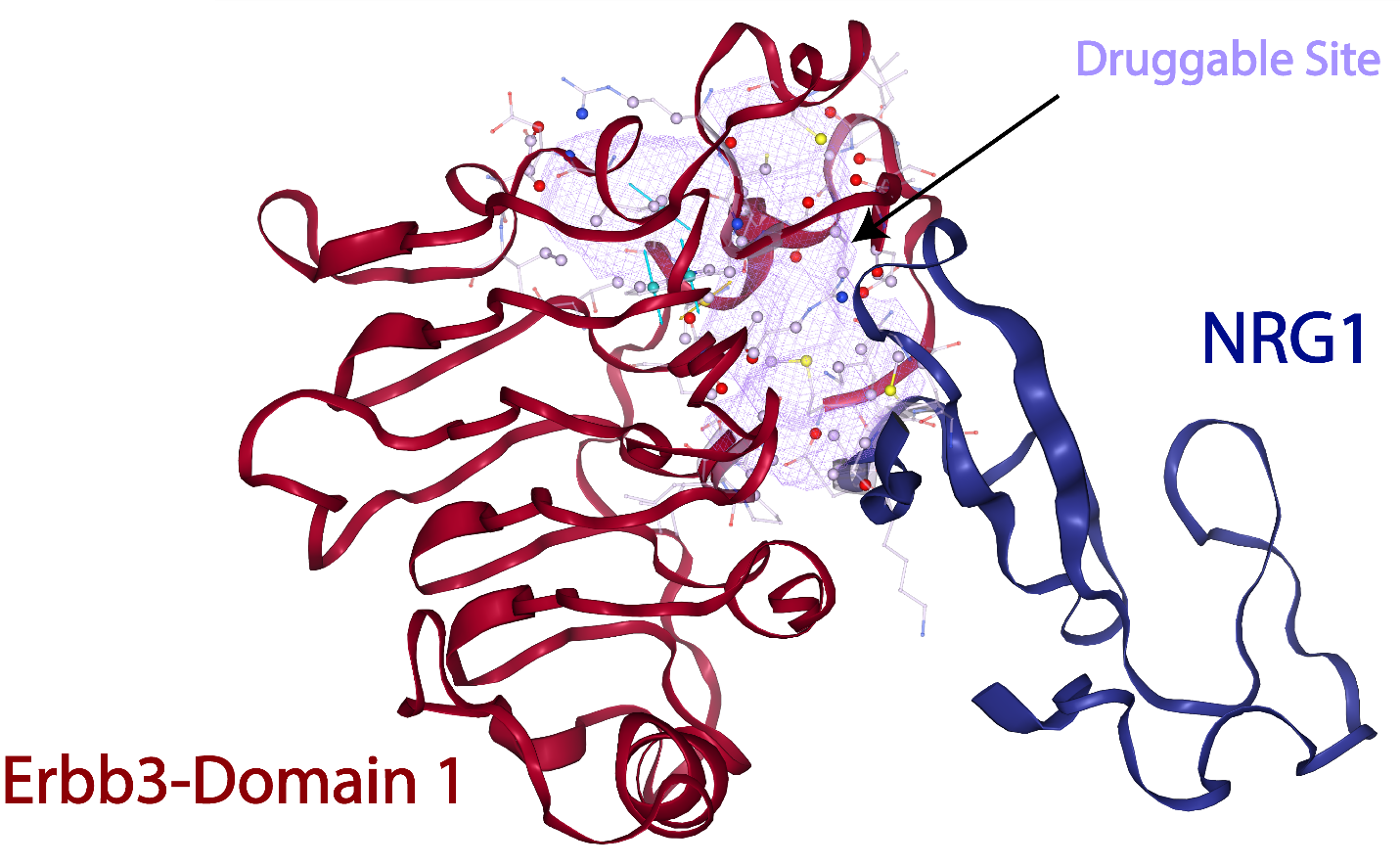


FigureS1: The druggable cavity pocket (purple) of NRG1(blue) with ERBB3 (red).
